# Development of an *In Vitro* Compartmentalization Screen for High-Throughput Directed Evolution of [FeFe] Hydrogenases

**DOI:** 10.1371/journal.pone.0015275

**Published:** 2010-12-06

**Authors:** James A. Stapleton, James R. Swartz

**Affiliations:** 1 Department of Chemical Engineering, Stanford University, Stanford, California, United States of America; 2 Department of Bioengineering, Stanford University, Stanford, California, United States of America; The Scripps Research Institute, United States of America

## Abstract

**Background:**

[FeFe] hydrogenase enzymes catalyze the formation and dissociation of molecular hydrogen with the help of a complex prosthetic group composed of common elements. The development of energy conversion technologies based on these renewable catalysts has been hindered by their extreme oxygen sensitivity. Attempts to improve the enzymes by directed evolution have failed for want of a screening platform capable of throughputs high enough to adequately sample heavily mutated DNA libraries. *In vitro* compartmentalization (IVC) is a powerful method capable of screening for multiple-turnover enzymatic activity at very high throughputs. Recent advances have allowed [FeFe] hydrogenases to be expressed and activated in the cell-free protein synthesis reactions on which IVC is based; however, IVC is a demanding technique with which many enzymes have proven incompatible.

**Methodology/Principal Findings:**

Here we describe an extremely high-throughput IVC screen for oxygen-tolerant [FeFe] hydrogenases. We demonstrate that the [FeFe] hydrogenase CpI can be expressed and activated within emulsion droplets, and identify a fluorogenic substrate that links activity after oxygen exposure to the generation of a fluorescent signal. We present a screening protocol in which attachment of mutant genes and the proteins they encode to the surfaces of microbeads is followed by three separate emulsion steps for amplification, expression, and evaluation of hydrogenase mutants. We show that beads displaying active hydrogenase can be isolated by fluorescence-activated cell-sorting, and we use the method to enrich such beads from a mock library.

**Conclusions/Significance:**

[FeFe] hydrogenases are the most complex enzymes to be produced by cell-free protein synthesis, and the most challenging targets to which IVC has yet been applied. The technique described here is an enabling step towards the development of biocatalysts for a biological hydrogen economy.

## Introduction

[FeFe] hydrogenase enzymes are very active hydrogen producers [1] but are extremely sensitive to oxygen, which is thought to diffuse through two putative gas channels in the protein to poison the H-cluster cofactor at the active site [2]. This sensitivity reduces the applicability of the enzymes in biotechnological hydrogen production schemes, for which they are otherwise very promising. Narrowing the gas channels may prevent oxygen from diffusing to the active site, but finding mutations that accomplish this is a difficult challenge. The failure of previous attempts at evolving oxygen tolerance suggests that multiple synergistic mutations may be required before any improvement is observed [3].

*In vitro* compartmentalization (IVC) is a technology with the potential to enable high-throughput screening of [FeFe] hydrogenase mutants. In IVC, extremely small aqueous droplets suspended in a continuous oil phase isolate individual mutant DNA molecules, forming independent emulsion cell-free protein synthesis (eCFPS) reactors. Analogous to cells in an *in vivo* screen, the droplets co-localize the gene, the mutant protein it encodes, and the products of the desired enzymatic activity [4]. Like other *in vitro* methods such as ribosome display [5] and mRNA display [6], IVC can accommodate very large mutant libraries and is free of the biases inherent in *in vivo* platforms. However, IVC is unique among high-throughput *in vitro* methods in its ability to screen for multiple-turnover catalytic activity [7]. Droplet-based technology is advancing rapidly as its potential for evaluating mutants [8], determining the effects of drug candidates on individual encapsulated cells [9], [10], and accelerating DNA sequencing [11], [12] becomes apparent. Combining IVC with microfluidic technology allows monodisperse emulsion droplets to be formed [13], mixed [14], split [15], merged [10], incubated, thermocycled [16], ordered, assayed for fluorescence [17], and sorted [18], all within the confines of a small chip.

Depending on the target of the directed evolution project, IVC can be configured as a selection (in which the mutant gene itself is generally the substrate for the desired activity or binding) or as a high-throughput screen in which fluorescence-activated cell sorting (FACS) is used to analyze and sort microbeads [8], [19] or water-in-oil-in-water (w/o/w) double emulsions [20], [21] on the basis of fluorescence linked to the desired activity. The power of FACS in directed evolution applications has previously been demonstrated by techniques such as yeast display [22] and bacterial surface display [23]. In the microbead display IVC method, mutant DNA and the protein it encodes bind to the surface of microbeads within emulsion droplets. The compartmentalization imposed by the droplets ensures that each gene and its encoded protein bind to the same bead. The resultant physical genotype-phenotype linkage is maintained following emulsion breakage and bead pooling. If the desired enzymatic activity generates a fluorescent product which can also bind to the surface of the beads, the beads can be sorted by FACS following recovery from the emulsion. Genes encoding positive mutants are then amplified from the sorted beads by PCR. Attachment of fluorescent products to beads has been achieved by caged-biotinylation of the fluorogenic substrate [24] or by generation of a radical that reacts with bead-bound proteins [19].

Recently, our laboratory demonstrated production of active [FeFe] hydrogenases in the cell-free protein synthesis reactions at the heart of IVC [25], [26], making the development of an IVC screen for oxygen-tolerant [FeFe] hydrogenases possible. Here we apply IVC to the directed evolution of [FeFe] hydrogenase I (referred to as CpI) from *C. pasteurianum*, a complex metalloenzyme containing multiple iron-sulfur electron transport clusters and an H-cluster catalytic prosthetic group composed of [4Fe-4S] and [2Fe-2S] clusters augmented by cyanide, carbon monoxide, and dithiolate ligands [26], [27]. We demonstrate an IVC system that links [FeFe] hydrogenase activity to the generation of a fluorescent signal on the surface of microbeads. Cell-free protein synthesis reactions within monodisperse emulsion droplets produce mutated CpI proteins, which bind to the surfaces of beads along with their encoding DNA templates. Following oxygen exposure, surviving [FeFe] hydrogenase mutants reduce the fluorogenic compound C_12_-resazurin, allowing detection by FACS. We demonstrate the system by enriching a mock library for beads bound to CpI DNA.

## Results and Discussion

### [FeFe] hydrogenase can be produced by emulsion CFPS

Previously we demonstrated the ability to produce and activate [FeFe] hydrogenases in bulk CFPS reactions. However, many proteins have been found to be incompatible with CFPS expression within emulsion droplets [28]. To test whether the helper protein-mediated synthesis and installation of the H-cluster prosthetic group could occur within emulsions, we added linear DNA templates encoding CpI to extracts of *E. coli* BL21 DE3 containing the three [FeFe] hydrogenase maturases [29] from *Shewanella oneidensis*, and emulsified the mixtures by stirring, vortexing, or extruding the aqueous phase into various oil/surfactant mixtures. Methyl viologen assays [30] detected hydrogenase activity in the collected products of the eCFPS reactions, indicating successful synthesis and activation of [FeFe] hydrogenases within the emulsion compartments.

### Resazurin derivatives as fluorescent sensors of hydrogenase activity

**Figure 1:**
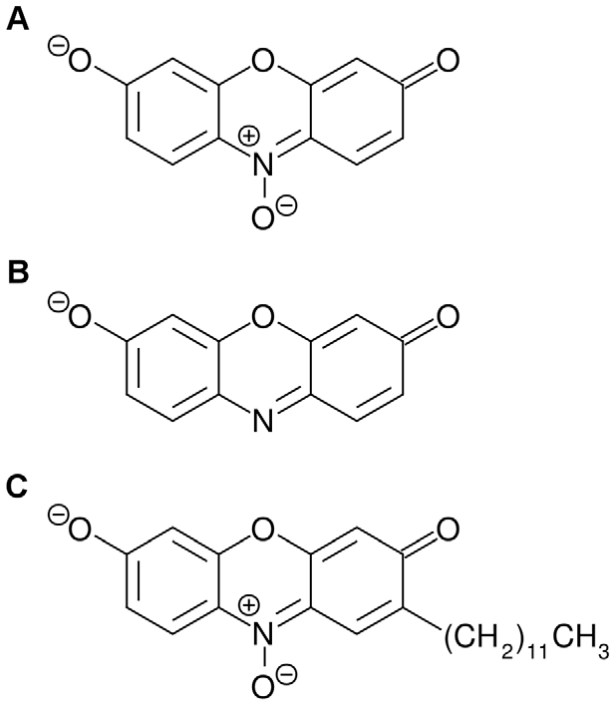
Resazurin and its derivatives. Resazurin (**A**) is irreversibly reduced to fluorescent resorufin (**B**) by electrons liberated from hydrogen gas by [FeFe] hydrogenase. C12-resazurin (**C**) is identical to resazurin except for an additional hydrophobic twelve-carbon tail.

Isolation of oxygen-tolerant mutants by FACS requires that a fluorescent signal develop in the presence of an active hydrogenase surviving oxygen exposure. We evaluated the ability of a number of redox-sensitive fluorophores to exchange electrons directly with hydrogenase. The only compatible fluorogenic molecule we identified was resazurin (Figure 1a, also known as AlamarBlue), which was irreversibly reduced to the fluorescent resorufin (Figure 1b) by electrons liberated from hydrogen gas by CpI.

Resazurin and resorufin are not suitable for flow cytometry because they quickly leak out of cells, and examination by fluorescence microscopy revealed that they also leaked out of our emulsion droplets. Fortunately, the resazurin derivative C_12_-resazurin (Figure 1c), which has the same fluorescent properties as resazurin, is much better retained by cells, and is marketed for use in FACS. We tested C_12_-resazurin and found that it was retained by our emulsion droplets much better than unmodified resazurin.

**Figure 2:**
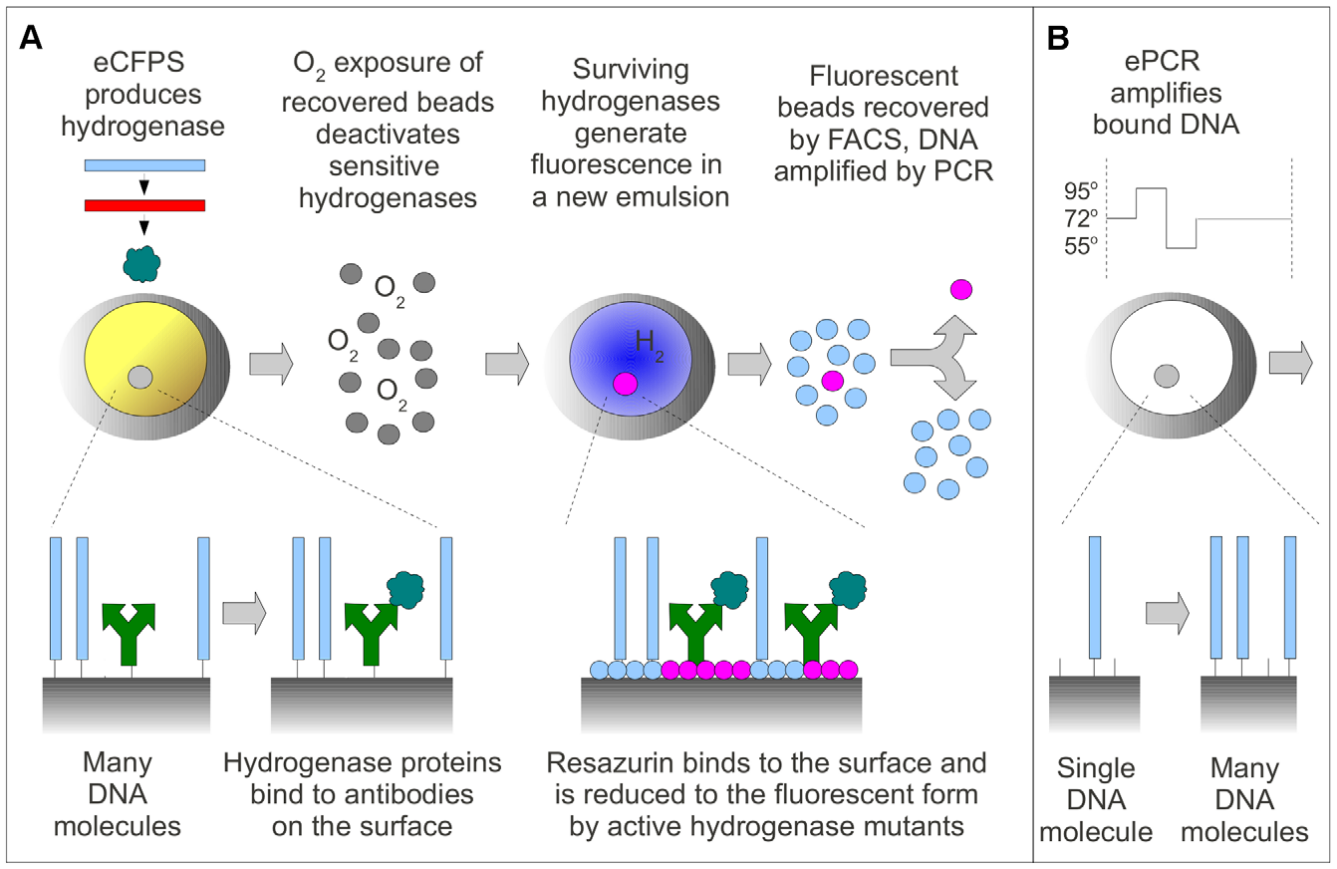
A schematic representation of the IVC screen protocol. The lower section of the diagram shows the molecules bound to the surface of the streptavidin-coated beads at each step. Blue rectangles represent template DNA, green split arrows represent biotinylated anti-HA antibodies, turquoise clouds represent 3xHA-tagged hydrogenase proteins, blue and magenta circles represent C_12_-resazurin and C_12_-resorufin. **A.** DNA-bound beads are incubated with biotinylated anti-HA antibodies, added to a cell-free protein synthesis mixture, and emulsified into an oil phase. Emulsion CFPS transcribes mRNA from the bound DNA templates and synthesizes HA-tagged hydrogenase proteins, which are bound by the antibodies. The beads are then recovered from the emulsion, washed, and exposed to oxygen to challenge the hydrogenase mutants. The beads are then re-emulsified, and C_12_-resazurin is delivered to the emulsion droplets. Mutant hydrogenases that survive oxygen exposure consume hydrogen and reduce C_12_-resazurin to fluorescent C_12_-resorufin, which adsorbs to the bead surface. The beads are recovered and sorted by FACS. Fluorescent beads are added to a PCR mixture and thermocycled to recover DNA encoding improved hydrogenase mutants. **B.** An emulsion PCR step amplifies unique mutant templates to amounts sufficient to result in strong fluorescent signals. Beads are first incubated with biotinylated primers (represented by short black lines) and less than one molecule of template DNA per bead, then added to a PCR mixture and emulsified. The emulsion is thermocycled, and the beads are recovered, washed, and screened with the procedure shown in **A**.

C_12_-resazurin is marketed as a cell viability assay reagent because active metabolic pathways reduce it to the fluorescent form. We found that it is also readily reduced by pathways active in our *E. coli* cell extract. Co-location of the cell extract and C_12_-resazurin within the same emulsion droplet would therefore result in indiscriminate production of the fluorescent product. To avoid this problem we adopted the microbead display strategy discussed earlier, which creates a physical genotype-phenotype linkage that allows the beads to be removed from emulsions, washed, resuspended in a new assay solution, and re-emulsified. Streptavidin-coated beads displaying biotinylated DNA templates and biotinylated anti-hemagglutinin (HA) tag antibodies were mixed into a CFPS reaction solution and emulsified into a continuous oil phase. Within the droplets, triply HA-tagged hydrogenase was synthesized, matured, and bound to the antibodies. The beads could then be broken out of the emulsion, washed, and re-emulsified for a separate fluorescence-generation reaction step. We delivered C_12_-resazurin to pre-formed emulsion droplets [31] to ensure that the reaction did not begin before the beads were compartmentalized. A schematic of the screening process is shown in Figure 2a.

C_12_-resorufin nonspecifically adsorbs to the surfaces of the polystyrene beads, allowing beads recovered from fluorescence-generation emulsions to be directly sorted by FACS. Beads coated with C_12_-resorufin can be washed and stored for weeks with little decay of the fluorescent signal. Switching from the 1 µm diameter beads used in previous reports to 5.6 µm diameter beads increased the surface area available to bind C_12_-resorufin, and dramatically improved signal resolution.

### Hydrogenase-bound beads can be sorted by C_12_-resorufin fluorescence

**Figure 3:**
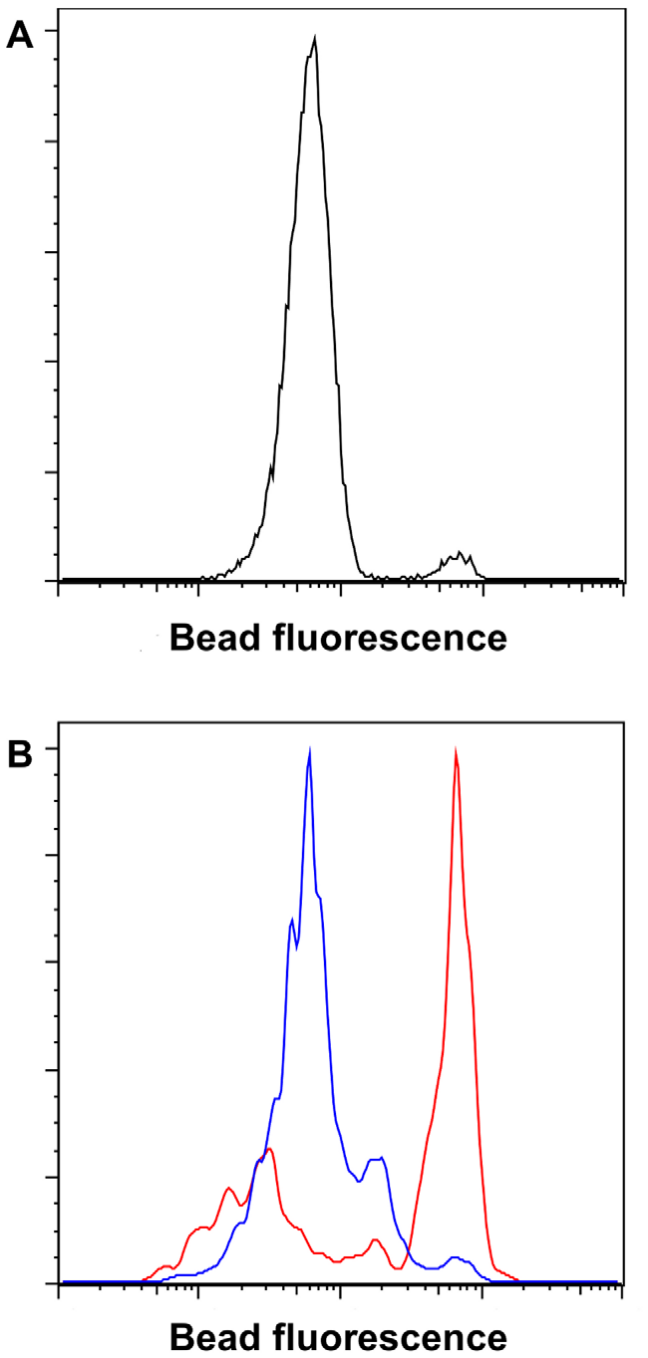
Beads can be sorted on the basis of C_12_-resorufin fluorescence linked to hydrogenase activity. **A.** The distinct peak separation between the two fluorescence populations after FACS analysis of a sample consisting of a mixture of beads with and without bound hydrogenase confirms that fluorescent C12-resorufin does not leak between emulsion droplets. The antibody-coated beads were incubated with a bulk CFPS reaction mixture synthesizing triply HA-tagged hydrogenase or with a template-free reaction mixture. The beads were then washed, mixed at a 1∶50 ratio, and subjected to the fluorescence generation emulsion step and subsequent FACS analysis. **B.** Decrease in bead fluorescence generation ability caused by deactivation of hydrogenase following incubation in a buffer containing ∼0.25 mM dissolved oxygen. Following hydrogenase production by eCFPS, the beads were recovered and washed. The sample was split in half, and one half was incubated in 1.5 mL of air-equilibrated 50 mM Tris-HCl pH 9 for 60 minutes. Both samples were then subjected to the fluorescence generation emulsion step and evaluated by FACS.

To test the feasibility of sorting beads on the basis of C_12_-resorufin fluorescence, we mixed beads coated with anti-HA antibodies and ∼1000 molecules of CpI DNA per bead with a 50∶1 excess of beads coated with antibodies but no DNA. The bead mixture was suspended in CFPS reaction solution, emulsified into oil, and incubated. Following protein synthesis the beads were recovered, resuspended in assay solution, re-emulsified, and incubated to allow antibody-bound hydrogenase to reduce C_12_-resazurin to C_12_-resorufin. Finally, the beads were recovered and analyzed for C_12_-resorufin fluorescence by FACS. In the fluorescence histogram of the single sample, two separate populations are easily distinguished (Figure 3a). The result confirms that the fluorescent C_12_-resorufin molecules were unable to leak between emulsion compartments during the fluorescence generation incubation, and that C_12_-resorufin fluorescence generated by hydrogenase activity can be used to identify active enzymes.

To confirm that exposure of wild-type hydrogenase to oxygen would decrease subsequent fluorescence generation, we incubated hydrogenase-coated beads in an air-equilibrated buffer solution. As expected, following oxygen exposure the ability of the beads to generate C_12_-resorufin fluorescence was diminished (Figure 3b).

### A microfluidic chip allows the creation of monodisperse emulsion droplets

**Figure 4:**
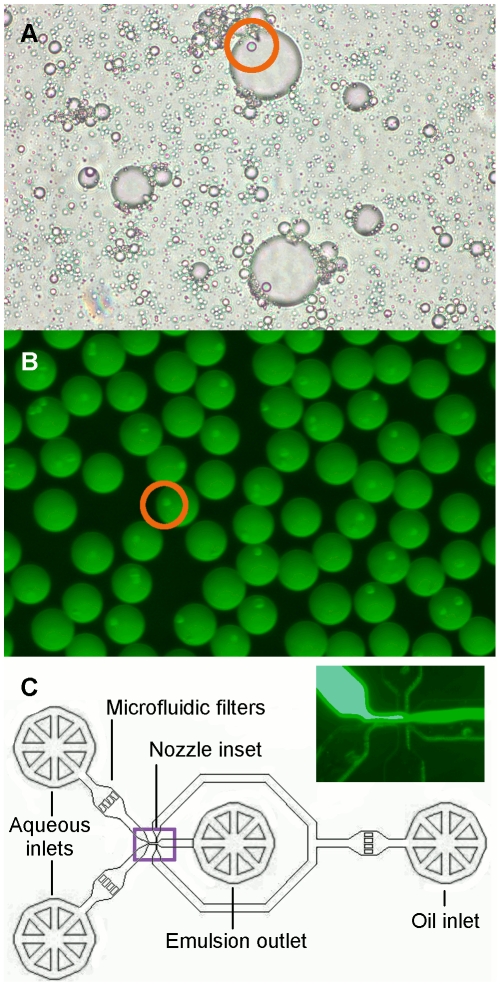
Creation of monodisperse emulsion droplets with a microfluidic device. **A.** A polydisperse water-in-oil emulsion created by stirring. Beads (5.6 µm, orange circle) encapsulated in droplets are visible. **B.** Monodisperse droplets produced with the microfluidic device. Two solution streams, one containing 5 mM fluorescein, met immediately upstream of the emulsification nozzle. The resulting droplets were evenly fluorescent, indicating that each contained an equal mixture of the two solutions and that the laminar solutions mixed within the droplets. Beads (5.6 µm, orange circle) are visible within the droplets. **C.** Design of a microfluidic chip for the production of monodisperse emulsion droplets. Circular structures indicate punch holes for insertion of the steel pins through which fluids enter and exit the chip. Aqueous phases enter on the left, oil enters on the right, and the emulsified product exits from the punch hole in the center. Microfluidic filter pillars prevent dust and other obstructions from clogging the nozzle. Main channels are 100 µm wide, and narrow to 20 µm. All features are 25 µm in height. Inset photograph shows the nozzle during the formation of the droplets pictured in **B**.

Emulsion droplets produced by bulk methods such as stirring are highly polydisperse (Figure 4a), potentially leading to uneven protein production across otherwise identical beads. To improve droplet monodispersity, we designed a flow-focusing microfluidic chip [32] capable of forming droplets at rates of several kilohertz (Figure 4c). The design of the chip was based on published microfluidic droplet generators. Two aqueous solutions, one consisting of the beads and the small molecule components of the eCFPS reaction mixture and the other primarily of diluted cell extract, were injected into the chip through separate ports. The solutions met immediately upstream of the emulsification nozzle, ensuring that transcription did not begin before the beads were compartmentalized within droplets. Experiments in which fluorescein was added to one of the two mixtures confirmed that the solutions evenly mixed within the droplets (Figure 4b), providing a complete and uniform eCFPS reaction mixture. The throughput of the chip, while much lower than that of bulk emulsification methods, is comparable to that of FACS, and therefore does not dramatically decrease the throughput of the overall process.

### Emulsion PCR amplifies a single template molecule to improve eCFPS yield

Though we observed a strong, saturated fluorescent signal when we attached ∼1000 DNA molecules to each bead, when we attached single DNA templates to the beads we found that the reduced numbers of enzyme copies that resulted were insufficient to generate significant fluorescence. Successful microbead display screens reported in the literature have incorporated mechanisms, such as a caged biotin, to keep the fluorogenic substrate soluble during the reaction period and only allow it to bind to the bead once the reaction is complete. In our system, C_12_-resazurin presumably binds to the bead immediately upon delivery into the aqueous compartment. We hypothesized that the bound hydrogenase enzymes were only able to reduce C_12_-resazurin molecules bound nearby on the bead surface, which neither exchanged with C_12_-resorufin in solution nor diffused along the surface of the bead.

To solve this problem, we amplified the single template copy using emulsion PCR (ePCR, Figure 2b), a technique which has recently seen extensive use in next-generation sequencing [9], [33], [34], genotyping [35], and directed evolution [36]. Biotinylated primers were bound to the beads in numbers small enough to preserve ample streptavidin binding sites for the downstream antibody binding step. Amplification by ePCR of the single DNA molecule attached to the bead increased the number of copies of template in the subsequent eCFPS reaction, thereby increasing hydrogenase production and eventual signal generation. Compartmentalization of the beads within emulsion droplets is necessary during the ePCR step to ensure that each bead only binds DNA amplified from the single template on its surface, and does not exchange DNA with other beads. Only after the addition of the ePCR step to the protocol were we able to measure fluorescent signals from beads originally bound to single molecules of DNA.

Since amplification efficiency is dependent on droplet size, we formed emulsions for ePCR in the microfluidic device. The amplification efficiency of ePCR drops markedly with increasing amplicon length [37], and very low yields have been reported for targets the size of the CpI expression template (∼2 kb). Still, template dilution experiments indicated that even a modest increase in the number of DNA copies displayed on the bead could allow a significant increase in protein expression. Because amplification of long templates has been shown to be more efficient in larger emulsion droplet volumes [38], we set the oil and aqueous phase flow rates to produce droplets about 30 microns in diameter.

An additional benefit of the ePCR step is improved efficiency of DNA recovery by PCR from sorted beads following FACS. Some fraction of the bead-bound genes are expected to be partially or completely degraded by nucleases during the eCFPS incubation. Following ePCR, it should be much more likely that at least one copy of each gene will remain competent for amplification.

The microbead display format adopted here is a very useful option for FACS analysis of IVC-generated fluorescent signals. However, caged-biotinylated fluorogenic substrates or other convenient methods to control fluorophore/bead binding are often unavailable. It thus seems likely that an ePCR step will prove useful in many future applications of IVC.

### Enrichment of beads binding hydrogenase DNA

To demonstrate the ability of the screen to identify beads bound to single molecules of hydrogenase DNA, we performed a test enrichment experiment. We incubated two sets of beads with single-molecule per bead levels of biotinylated DNA templates encoding either tagged CpI or a negative control protein, chloramphenicol acetyltransferase (CAT). Both templates contained the same homoprimer [39] annealing sites for PCR amplification. We mixed the CAT and CpI beads at a 20∶1 ratio, performed the three emulsification steps of the IVC screen protocol (omitting the oxygen exposure step), and sorted the recovered beads by FACS. Low- and high-fluorescence beads were sorted and collected in separate tubes.

**Figure 5:**
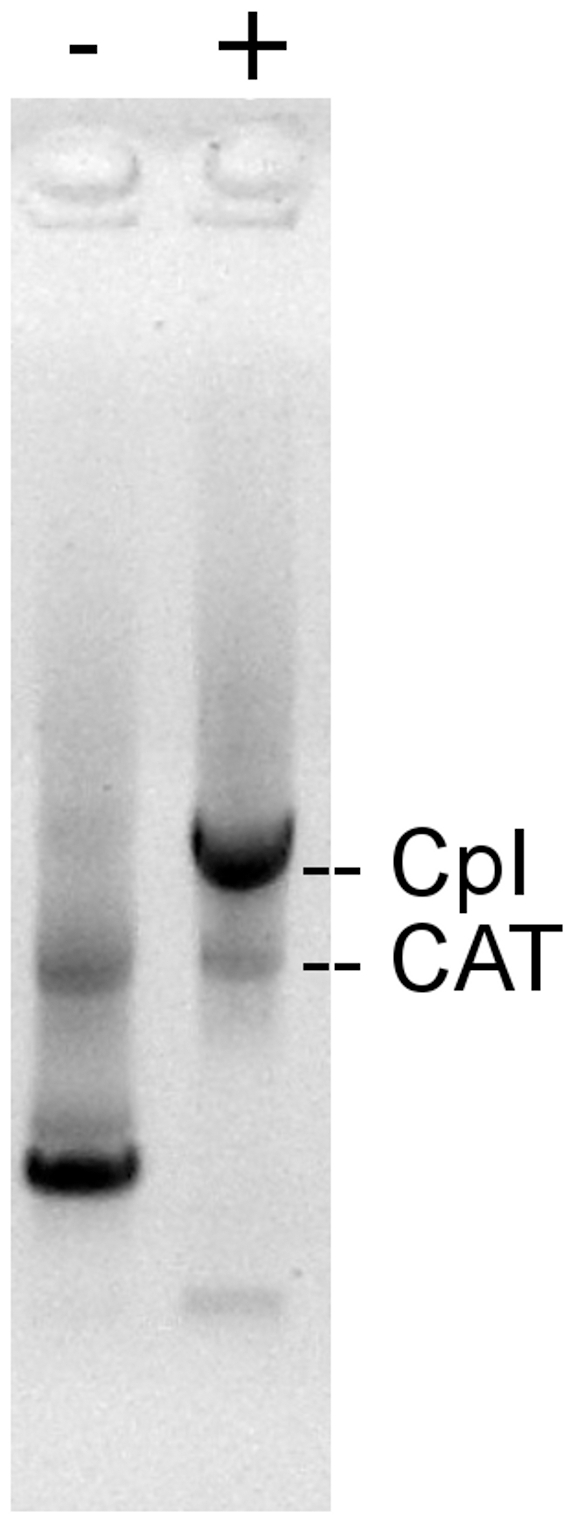
Enrichment of beads bound to single molecules of hydrogenase DNA. Beads displaying CAT DNA and beads displaying CpI DNA were originally mixed in a 20∶1 ratio. Comparison of the PCR amplification products from nonfluorescent (−) and fluorescent (+) sorted beads by agarose gel electrophoresis indicates that the more fluorescent population is enriched in CpI-bound beads. Nonspecific amplification products are visible in both lanes.

Amplification of the DNA on the sorted low-fluorescence beads gave rise to a bright CAT gene band and a very light CpI gene band, while the high-fluorescence beads yielded a bright CpI gene band and a light CAT gene band (Figure 5). This enrichment of CpI DNA indicates that the IVC screen was able to identify and sort beads initially bound to single molecules of DNA encoding an active [FeFe] hydrogenase. No enrichment was observed when the ePCR step was omitted from the protocol.

The success of the enrichment experiment indicates that [FeFe] hydrogenase mutants can be screened in an extremely high-throughput fashion by *in vitro* compartmentalization using C_12_-resazurin to link enzyme activity to the development of a florescent signal. However, further work will be required before screening of true mutant libraries can begin. The enrichment experiment described above used two populations of beads that were incubated with DNA separately and then mixed. A more realistic scenario is one in which the DNA is mixed before incubation with beads. Enrichment of hydrogenase DNA from mixtures with excesses of decoy DNA much larger than 20∶1 must also be demonstrated.

We also tested the platform described here with a second [FeFe] hydrogenase, HydA1 from *Chlamydomonas reinhardtii*, and confirmed its compatibility with both eCFPS and C_12_-resazurin. While this IVC screen was developed with the goal of discovering oxygen-tolerant hydrogenase mutants, it is actually a screen for hydrogenase activity, useful for screening for mutants with any of a number of industrially important characteristics, such as thermostability (by incubating beads at high temperatures between the eCFPS and C_12_-resazurin emulsion steps), tolerance for conditions such as might exist in a fuel cell, and so on. Hydrogenases with any of these phenotypes can be screened for with only minimal modifications to the platform described here. The throughput of IVC (thousands of beads per second on modern FACS machines) represents a dramatic improvement over traditional 96-well microtiter plate-based screens, and the methods described here provide a way forward towards the discovery of hydrogenase mutants with the potential to revolutionize the world's energy economy.

## Materials and Methods

### DNA preparation

The DNA template encoding C-terminally triply HA-tagged templates was created by overlap extension PCR using the pK7-CpI plasmid [26] with the following primers, which annealed outside the T7 RNA polymerase promoter and terminator and contained extensions for the addition of the TR homoprimer sequence [40] (5′-CCATTCATTAATGCACATAACTAT-3′) and the 3xHA tag: 5′-CCA TTC ATT AAT GCA CAT AAC TAT TCC AAC GAG TTC GCG GCC GCT TAG GCA CCC CAG GCT TTA C-3′, 5′-GGC ATA GTC TGG GAC GTC ATA TGG ATA TGC ATA GTC CGG AAC ATC GTA TGG GTA TTT TTT ATA TTT AAA GTG CAG GAT TTC-3′, 5′-CCA TTC ATT AAT GCA CAT AAC TAT TCC AAC GAG TTC GAC GAG CGT CAG CTT GCA TGC CCT GCA GCT-3′, 5′-TAT CCA TAT GAC GTC CCA GAC TAT GCC TAC CCG TAT GAC GTG CCA GAT TAC GCG TAA TAA TAA TTT TTT TAA GGC AGT TAT TGG TG-3′. The CAT linear template was created from the pK7-CAT plasmid in the same way. The linear templates were re-amplified with biotinylated TR homoprimer to yield constructs with biotin groups at both ends.

Following purification with PCR cleanup kits (Qiagen, Valencia, CA), DNA concentrations were measured with the Quant-It dsDNA broad-range assay on a Qubit fluorimeter (Invitrogen, Carlsbad, CA) following the manufacturer's protocol. The solutions were then diluted in water or TE buffer (10 mM Tris-HCl pH 8, 1 mM EDTA) to 10^7^ molecules/µL.

### Bead preparation

2×10^7^ 5.6 micron diameter streptavidin-coated beads (CP01N, Bangs Laboratories, Fishers, IN) were washed with DNA binding buffer (1 mM NaCl, 10 mM Tris-HCl, pH 7.5), resuspended in the same, and incubated with 0.3–1 molecule of biotinylated linear DNA template per bead for 16 hours at room temperature with rotation to prevent settling. The beads were then incubated in DNA binding buffer with 3 million biotinylated reverse primers per bead for 2 hours, and washed in PBS.

### Microfluidic chip design and preparation

A PDMS microfluidic flow-focusing emulsification device with the design shown in Figure 4c was fabricated by the Stanford Microfluidics Foundry (http://thebigone.stanford.edu/foundry/index.html). The chip was bonded to a glass substrate by PDMS-PDMS bonding. The device was pre-treated with Aquapel [41] (PPG Industries, Pittsburgh, PA) to render the channels hydrophobic and ensure that the oil phase preferentially wetted the surface. The chip was designed with a uniform 25 µm feature height. Channels narrow from a width of 100 µm to 20 µm at the nozzle.

### Emulsion PCR

Following incubation with DNA templates and biotinylated ePCR primers as described above, the beads were resuspended in a mixture of 1X Pfu buffer, 0.2 mM each dNTP, 0.1 µM TR homoprimer, 0.1% Tween 20, and 0.2 U/µL Pfu Turbo polymerase (Stratagene, La Jolla, CA) at a bead concentration of 5×10^4^/µL.

The PCR mixture and an oil phase consisting of Fluorinert FC-40 (Sigma Aldrich, St Louis, MO) with 2% w/w PFPE-PEG block copolymer surfactant shown to be compatible with both PDMS chips and eCFPS [42] (provided courtesy of Raindance Technologies, Lexington, MA) were forced into the inlets of the microfluidic chip described above at constant flow rates with syringe pumps. The second aqueous inlet was blocked with a clamped tube. An aqueous flow rate of 6 µL/min and an oil flow rate of 50 µL/min generated monodisperse droplets approximately 30 µm in diameter.

The emulsion was distributed in 50 µL aliquots into thin-walled PCR tubes and heated at 95°C for 1 minute, followed by 50 cycles of 95°C for 10 seconds, 55°C for 30 seconds, and 72°C for 2 minutes. The thermocycled emulsions were collected into one tube and broken by addition of a volume of A104 emulsion destabilizer (provided by Raindance Technologies, Lexington, MA) equal to 10% of the volume of emulsified aqueous phase, followed by gentle stirring with a pipette tip and recovery of the upper aqueous phase. Beads were then incubated in PBS with 200,000 biotin-conjugated rabbit anti-HA antibodies per bead (Immunology Consultants Laboratory, Inc., Newberg, OR) for two hours at room temperature.

### Cell-free Protein Synthesis

Inside an anaerobic glove box the cell-free extract harboring the three [FeFe] hydrogenase helper proteins HydE, F, and G was reconstituted [26] by incubation at room temperature with 0.5 mM ferrous ammonium sulfate and 0.5 mM Na_2_S, and then spun at 10,000 g until all precipitates were removed. The following two solutions were prepared (concentrations given are those in the final CFPS mixture, which consisted of an equal mixture of Solution A and B): Solution A consisted of a small molecule substrate mixture [25] and 0.1% Tween 20 (Sigma Aldrich, St Louis, MO) to prevent bead aggregation. Solution B consisted of 6.7 µg/mL bacteriophage lambda Gam protein (to inhibit RecBCD exonuclease in the extract), 0.1 mg/mL T7 RNA polymerase, and 25% v/v *E. coli* cell extract. Gam protein and T7 RNA polymerase were expressed and purified in house.

Solution A was added to washed, pelleted beads and vortexed to give a suspension of 10^5^ beads/µL. Solutions A and B were loaded into separate Hamilton gastight syringes. The FC-40/surfactant oil phase described above, having been stored in the glove box to remove oxygen, was loaded into a third, larger syringe.

The microfluidic chip described above was used to generate monodisperse emulsions. Flow rates of 3 µL/min of each of the two aqueous phases and 50 µL/min of the oil phase produced monodisperse droplets approximately 30 µm in diameter. Emulsification was carried out outside the glove box on an inverted microscope. The emulsified mixture was collected into an airtight vial (containing 500 µL buffer to dilute and thus prevent protein synthesis from taking place in any cell-free mixture that avoids emulsification) and returned to the glove box immediately upon completion. The emulsified reactions were incubated at room temperature for 6–16 hours. Following this incubation the emulsion was broken with A104 emulsion destabilizer as before.

When emulsions were formed by bulk methods, 60 µL of the combined CFPS mixture above (omitting Tween 20) was added to the pelleted beads, mixed, and immediately emulsified into 500 µL cold microbiology grade light mineral oil containing 1.8% v/v Span 80 and 0.2% v/v Triton X-100 (all from Sigma Aldrich, St Louis, MO) surfactants. Emulsions were formed by stirring the mixture in the bottom of a 2 mL round-bottom cryovial with a 3 mm×8 mm magnetic stir bar at 600 rpm for 7 minutes, briefly vortexing at maximum speed after each minute. Alternatively, eCPFS emulsions were prepared by extrusion, with an oil phase of mineral oil with 1.8% v/v Span 80 and 0.2% v/v Triton X-100, or of decane with 1% w/v Span 60 and 1% w/v cholesterol (all from Sigma Aldrich, St Louis, MO). The beads were suspended in 50 µL of CFPS solution and extruded through a 19 mm track-etch polycarbonate membrane with 14 µm pores (GE Osmonics, Minnetonka, MN) into 200 µL of oil using a hand extruder (Avanti Polar Lipids, Alabaster, AL). After a total of 15 passes through the membrane the emulsion was dispensed into a 1.5 mL tube. The emulsified CFPS reaction droplets were incubated at room temperature for 16 hours. After the incubation, the emulsion was centrifuged to settle the droplets to the bottom of the tube, and the clarified oil phase was removed. 400 µL of 10 mM Tris-HCl pH 8 with 0.5% v/v Tween 20 (Sigma Aldrich, St Louis, MO) was added, and the emulsion was centrifuged at 10000 g for 3 minutes to break the beads out of the destabilized droplets, vortexed, and centrifuged for another three minutes. This resulted in a pellet of beads at the bottom of the tube, buffer in the middle, and a thick oil phase at the top. The pelleted beads were aspirated from the bottom of the tube with a pipette and washed in a new tube with 200 µL 50 mM Tris-HCl pH 9, then resuspended in the same buffer.

### Oxygen Exposure

For oxygen exposure experiments, a 1.5 mL tube completely filled with 10 mM Tris-HCl pH 8 equilibrated with air was moved into the anaerobic chamber. Suspended beads were added to the tube and incubated for 60–90 minutes before being pelleted by centrifugation and washed with anaerobic buffer.

### Fluorescent signal generation

Oxygen-exposed beads were suspended in 30 µL of 50 mM Tris-HCl pH 9 and re-emulsified by extrusion as described above into 300 µL of mineral oil mixed with 3% v/v Abil EM90 surfactant (Evonik Degussa, Essen, Germany), which has been reported to perform well in redox-sensitive emulsion applications [43]. 0.3 µL of a 10 mM solution of C_12_-resazurin (Invitrogen, Carlsbad, CA) in DMSO was added to the emulsion, which was vortexed for 30 seconds to deliver the C_12_-resazurin to the droplets. The emulsion was then incubated anaerobically in the dark at room temperature for 2–16 hours. Following the incubation, the emulsion was removed from the anaerobic chamber and centrifuged at 20,000 g for 1 minute, and the clarified excess oil phase was removed. 400 µL of 10 mM Tris-HCl pH 8 with 0.5% v/v Tween 20 (Sigma Aldrich, St Louis, MO) was added to disrupt the emulsion, which was then centrifuged at 20,000 g for three minutes, vortexed, and centrifuged for another three minutes. The pelleted beads were aspirated from the bottom of the tube with a pipette and washed in a new tube with 200 µL 50 mM Tris-HCl pH 9 (the pH at which C_12_-resorufin is maximally fluorescent), then resuspended in the same buffer and analyzed by FACS.

### FACS analysis and sorting

Beads were analyzed and sorted using various flow cytometers at the Stanford Shared FACS Facility. A dye laser tuned to 570 nm was used for excitation, and emission was collected through a 605/40 nm filter. Beads were analyzed at rates of 500–1000 events per second.

### Recovery of mutant DNA by PCR

Unsorted and sorted beads were amplified with Phusion DNA polymerase (Finnzymes, Espoo, Finland) using a single-molecule PCR protocol described previously [25].

**Note on proprietary reagents:** it is the policy of Raindance Technologies to provide commercially unavailable reagents to academic researchers at no cost.
